# Developing a method to assess fidelity to a complex vocational rehabilitation intervention in the FRESH trial: a feasibility study

**DOI:** 10.1186/s40814-022-01111-2

**Published:** 2022-07-29

**Authors:** Jain Anne Holmes, Joanna Clare Fletcher-Smith, Jose Antonio Merchán-Baeza, Julie Phillips, Kathryn Radford

**Affiliations:** 1 Faculty of Medicine and Health Sciences, Centre for Rehabilitation and Ageing Research, B Floor, Medical School, Queen’s Medical Centre, The University of Nottingham, Nottingham, NG7 2UH UK; 2 Faculty of Health Science and Welfare, University of Vic-Central University of Catalonia (UVIC-UCC), 08500 Vic, Spain

**Keywords:** Implementation, Complex intervention, Fidelity, Adherence, Moderating factors, Vocational rehabilitation, Brain injury, Mixed methods

## Abstract

**Background:**

Determining whether complex rehabilitation interventions are delivered with fidelity is important. Implementation fidelity can differ between sites, therapists delivering interventions and, over time, threatening trial outcomes and increasing the risk of type II and III errors. This study aimed to develop a method of assessing occupational therapists’ fidelity to deliver a complex, individually tailored vocational rehabilitation (VR) intervention to people with traumatic brain injury (TBI) and assess the feasibility of its use in a randomised controlled trial.

**Methods:**

Using mixed methods and drawing on the intervention logic model, we developed data collection tools to measure fidelity to early specialist TBI VR (ESTVR). Fidelity was measured quantitatively using intervention case report forms (CRF), fidelity checklists and clinical records. Qualitative data from mentoring records, interviews with intervention therapists, participants with TBI, employers and NHS staff at trial sites explored moderators of implementation fidelity. The conceptual framework of implementation fidelity (CFIF) guided measurement and analysis of and factors affecting fidelity. Data were triangulated and benchmarked against an earlier cohort study.

**Results:**

Fidelity to a complex individually tailored VR intervention could be measured. Overall, OTs delivered ESTVR with fidelity. Different fidelity measures answered different questions, offering unique insights into fidelity. Fidelity was best assessed using a fidelity checklist, intervention CRFs and clinical notes. The OT clinical notes and mentoring records were best at identifying fidelity moderating factors. Interviews added little insight into fidelity moderating factors over and above mentoring or clinical records. Data triangulation offered a comprehensive assessment of fidelity, highlighting limitations of measurement methods and learning for future trials but was resource intensive. Interviews, fidelity visits and analysing clinical notes were also resource intense. Comparing fidelity data to a benchmark and using CFIF as a framework for organising the fidelity assessment helped.

**Conclusions:**

OTs delivered the VR intervention with fidelity. A fidelity checklist and benchmark plus mentoring may offer a practical and effective way of measuring fidelity and identifying fidelity moderating factors in trials of complex individually-tailored rehabilitation interventions. Mentoring provided real-time indicators of and reasons for fidelity deviations. These methods require further evaluation.

**Trial registration:**

ISRCTN Registry, ISRCTN38581822 (Registered: 02/01/2014).

**Supplementary Information:**

The online version contains supplementary material available at 10.1186/s40814-022-01111-2.

## Background

Every year, traumatic brain injury (TBI) affects some 69 million people worldwide [1], including 160,000 in the UK [2]. TBI can result in impaired mobility, cognition, social skills and difficulty managing emotions [3, 4], which can affect return to work [5]. Return to work (RTW) is an important rehabilitation goal following TBI [6], yet only a mean of 41% (range 0–85%) of TBI survivors working before their injury are employed 1 and 2 years later [7]. Employment provides economic security and supports physical and psychosocial health but dedicated vocational rehabilitation services that support RTW are rare, and evidence on their effectiveness limited [8, 9].

Vocational rehabilitation (VR) defined as, “a multi-professional approach…provided to individuals of working age with health-related impairments, limitations, or restrictions with work functioning and whose primary aim is to optimise work participation” [10] is a complex rehabilitation intervention. It involves numerous components targeting work behaviours in people with TBI and their employers and requires specialist knowledge and skills on the part of the service provider [11]. It is also individually tailored to patients’ needs, circumstances, and jobs. Such individualised approaches make it harder to be certain that an intervention is delivered as intended or ‘with fidelity’.

Fidelity, defined as, “the degree to which...programs are implemented...as intended by the program developers” [12], is a moderator of successful patient and outcomes [13–15]. In rehabilitation trials, a lack of fidelity could undermine the trial results, adversely affecting outcomes or increase the risk of type III error [12].

Few studies have assessed the fidelity of occupational therapy interventions in clinical trials. In a review of fidelity in occupational therapy, Borrelli et al (2005) applied the treatment fidelity measure retrospectively to 17 studies with an experimental treatment and found mixed levels of fidelity. The authors concluded their findings may have been affected by a failure to report use of or design of fidelity strategies [16]. Another review evaluated the properties of fidelity measures used in occupational therapy studies. The authors found eight measures that were moderately thorough in their coverage of fidelity measurement and concluded that further research was required to develop novel measures for occupational therapy interventions [17].

In a trial of an activities of daily living intervention delivered by OTs in care homes, Masterson-Algar (2014) [18] found that to deliver the intervention with fidelity required OTs to learn how to deliver it in the context of the trial over time. They recommended the use of multiple data sources to determine whether the intervention was delivered as intended and an adapted Conceptual Framework for Implementation Fidelity (CFIF) [19] to guide fidelity measurement [18].

In a study to develop an occupational therapy protocol to improve depression and functional outcomes in older adults, adequate training to deliver the intervention alongside monitoring to support delivery was found to be important in demonstrating OTs’ adherence to key components [20].

Whilst observational methods and audio-recording, where a third-party checks whether selected intervention components are delivered, are regarded as the gold standard [21], they can be resource intense [22] and impractical when the intervention involves multiple recipients and delivery occurs in multiple contexts over a prolonged period. Other practically implementable methods include checklists or a benchmark to against which to measure adherence [23] [19].

A systematic review of barriers and facilitators affecting complex rehabilitation delivery in research identified factors that could potentially affect fidelity [24], which warranted further exploration. They included, patient needs and resources, readiness for implementation, facilitation strategies and participant responsiveness.

We developed a method of assessing occupational therapists’ fidelity to deliver early TBI specialist vocational rehabilitation intervention (ESTVR) a complex VR intervention delivered in the English National Health Service (NHS) and tested the feasibility of its use in the Facilitating Return to work through Early Specialist Health-based interventions (FRESH) trial [25].

### Aim

The aim is to develop and test a method of assessing fidelity to deliver a complex vocational rehabilitation intervention (VR) by occupational therapists in the FRESH feasibility RCT.

### Research questions

Can we develop a method of assessing occupational therapists’ fidelity to deliver early TBI specialist VR (ESTVR)?

Can fidelity to ESTVR be measured?

Can we identify the factors affecting fidelity to ESTVR?

## Methods

In the FRESH trial [25], 78 traumatic brain injured participants were randomly allocated to receive ESTVR from OTs trained in intervention delivery, in addition to their usual NHS rehabilitation (intervention group) or usual NHS rehabilitation alone (control group) over 12 months. The primary outcome was participants’ work status, defined as a minimum of an hour per week of paid or voluntary work, analysed at 12 months using an intention-to-treat approach.

Fidelity was measured as part of an embedded mixed methods process evaluation [25, 26] and involved multiple methods. The conceptual framework CFIF [19] was used to guide both the measurement of fidelity and understand factors affecting its delivery. The CFIF structure facilitated the development of measurement tools, including adapting an existing fidelity checklist [23], subsequently recommended by other scholars [27–29] and types of pertinent data to collect [23, 30].

Data collection was longitudinal illustrated in the flow diagram from the FRESH study (Additional file 1). Quantitative process data consisted of content of treatment records, fidelity checklists, mentoring records and clinical occupational therapy records and qualitative data collection methods included interviews, intervention and mentoring records (see Table 1). Both types of data enabled evaluation of the intervention process implementation and its fidelity during the study as subsequently recommended by Toomey, Hardeman [27].

**Table 1:** Data collection tools and timeframes for data collection

Tool	Timeframe	Data type	CFIF construct	Details	Data usage
Intervention CRF	Continuous recording; collected at end of intervention	Quantitative	Adherence	Quantity of components (10-min units) delivered per session, direct participant activity (face-to-face, telephone), travel, indirect activity (e.g. session preparation)	Triangulated with clinical record
Clinical notes	As above	Quantitative and qualitative	Adherence and moderating factors	Description of intervention plus evidence of correspondence	Triangulated with intervention CRF
Fidelity checklist	Quarterly at monitoring visits	Quantitative and qualitative	Adherence and moderating factors	Oversight per OT, extent of components delivered (always to never), moderating factors affecting delivery. Advise OTs	Triangulated with intervention CRF, clinical record, mentoring CRF.
Mentoring CRF	Monthly	Qualitative	Adherence and moderating factors	Intervention summary provided by each OT, details factors affecting delivery and potential solutions	Triangulated with intervention CRF, clinical record, fidelity checklist
Interview with OTs	Start of intervention delivery and end of delivery	Qualitative	Moderating factors	Addressed acceptability of the intervention, the factors affecting delivery and solutions to overcome barriers	Triangulated with mentor CRF, clinical record
Interview with PWTBI, their employers and NHS staff	End of intervention delivery	Qualitative	Moderating factors	Addressed acceptability of the intervention, the factors affecting delivery and solutions to overcome barriers	Triangulated with mentor CRF, clinical record

Continuous = OT completed the CRF and clinical record after each session. CFIF adherence includes intervention content, coverage, frequency and duration of intervention; CFIF moderating factors include participant responsiveness, intervention complexity, strategies to facilitate implementation, quality of delivery, recruitment and context

Feasibility was determined by (a) monitoring the amount of data collected and resources required to collect and analyse them and (b) determining which methods measured fidelity with accuracy and identified factors affecting fidelity most efficiently.

### Study participants

Participants were patients recruited to the FRESH feasibility randomised controlled trial (RCT) who were randomised to receive the ESTVR intervention. Inclusion criteria were people aged 16 years or above, admitted to one of three major trauma centres for 48 h or more, with a new TBI (within 8 weeks) and who were in paid or unpaid work or full-time education prior to injury. Full eligibility criteria in the FRESH RCT are explained elsewhere [31]. Clinical records and intervention session case report forms (CRFs) were collected for every intervention participant (*n* = 38). Purposive sampling was used to identify and recruit five participants for telephone interviews from each site with a range of demographics and TBI severity who had received the intervention (*n* = 15).

The five OTs who delivered the intervention were Health and Care Professions Council registered with expertise in VR. OTs attended two days of training, plus an additional day 6 months after intervention delivery commenced. Training was delivered by a team of four OTs with expertise in VR, TBI and research. Training was supplemented by an intervention manual and one-hour of monthly individual mentoring by a member of the training team to support implementation during the intervention delivery period. OTs could contact their mentor for advice when required. This is described elsewhere [32].

Employer participants included line managers, human resource professionals, or occupational health professionals of patient participants in employment or teaching staff linked to participants in full-time education. A convenience sample of 15 employers were recruited (five from each site). Only employers of participants with TBI (PwTBI) randomised to receive the intervention were eligible.

NHS staff participants at each site included those who, in their usual role, were involved in managing, commissioning, or delivering TBI rehabilitation. A convenience sample of 15 NHS staff were recruited (five from each site).

The VR intervention described in detail elsewhere [25] was delivered by OTs to TBI participants randomised to receive the intervention. The primary focus of the intervention was preventing job loss and optimising work outcomes. The intervention started within eight weeks of injury and lasted up to 12 months. The logic model for the FRESH intervention is described in Additional file 3.

### Data collection

Data were collected across all CFIF constructs to enable comprehensive analysis. Table 1 reports each data collection tool, when data was recorded and collected, data type, related CFIF construct, further details about the tool and data that were triangulated for agreement or disagreement. Quantitative data related to CFIF adherence (intervention content, coverage, frequency and duration). Qualitative data explained moderators of fidelity according to CFIF (participant responsiveness, intervention complexity, strategies to facilitate implementation, quality of delivery, recruitment, and context). Data collection occurred between January 2014 and January 2016. An independent researcher (AMB) triangulated data from the different tools to verify data for example ensuring intervention CRFs agreed with clinical records and identify if data were missing.

### Data collection tools

OTs were asked to record the content of each VR session using an intervention CRF (Additional file 2 [25]) adapted from Phillips [33]. The CRF recorded each component of the intervention and was adapted to ESTVR by ensuring that it matched the ESTVR logic model. Only one component ‘family support’ was added. OTs were asked to maintain ‘clinical notes’ following a format used in their employing organisation. This typically included the aim of the session, what occurred, clinical decision making and plan for the next session.

We developed a fidelity checklist (Additional file 3 [25, 34]) to measure OTs adherence to core intervention components and processes identified in the ESTVR logic model (Additional file 4 [25]). This was informed by an observational checklist used by Hasson [23]. Adherence to each item was rated on a 5-point ordinal scale: ‘always’, ‘often’, ‘sometimes’, ‘seldom’ or ‘never’ (where ‘always’ scored 1 and ‘never’ scored 5). Factors affecting intervention delivery were recorded free text alongside. Guidance notes were developed to support implementation and explain adherence and moderators.

Each OT received four fidelity check visits from a post-doctoral research OT (JP) with clinical expertise in the trial intervention. OTs provided anonymised copies of all clinical notes and intervention CRFs prior to the visit. The research OT and intervention delivery OT met to discuss the intervention delivered to participants and the research OT completed the checklist. Checklist data were transferred to Excel by a member of the study team (JMB). Completed fidelity checklists were discussed by the study team and issues affecting adherence to intervention delivery were translated into topics for skill-building during mentor sessions.

Each mentoring session was recorded on a CRF developed for this purpose (Additional file 5 [25, 34]). The form recorded the OT and mentor, date of session, time spent in mentoring and mode of session, e.g. telephone, face-to-face. Additionally, topics addressed during mentoring were documented. Topics typically included participant recruitment, study documentation completion, implementation of the intervention, clinical decision-making about participants and potential or actual serious adverse events. The amount of time spent in and content of any additional mentoring support provided by email and phone calls was recorded.

Interviews with OTs were conducted early after training and later to capture the OTs varying experience of delivering the intervention. People with TBI and their employers and NHS staff were interviewed at the end of the intervention. Interviews followed a topic guide (reported elsewhere [25]) informed by the theoretical constructs of CFIF to capture qualitative data on factors affecting implementation fidelity. Interviews took place by telephone and lasted approximately 45 min. They were digitally recorded, fully transcribed, cleaned and the data was uploaded to SQR Nvivo software for analysis.

### Data analysis

The intervention logic model and a ‘fidelity’ benchmark were used to guide data analysis and interpretation. Durlak and DuPre (2008) [14] indicated, in their meta-analysis of 542 interventions between 1976 and 2006, that outcomes were effective when interventions were delivered with 60–80% fidelity. They advised that variation in fidelity across sites should be reported because overall fidelity can mask expected variation.

The benchmark used for this comparison was Phillips [33] description of an early VR intervention for people with TBI, which informed the development of the FRESH intervention. Quantitative data about the proportion of components delivered by the OTs were compared to data provided by Phillips [35] to illustrate fit with the core VR components identified by Phillips.

Fidelity checklists were completed after each fidelity visit and after triangulating data, e.g. frequency of sessions by locating this evidence, if available, in clinical notes, mentoring records and intervention CRFs. The five-point scale provided an overall indication of fidelity. These data sources were compared to identify any variations in fidelity and discrepancies between data sources. For example, the date of discharge from the VR intervention was missing on an intervention CRF but was recorded in the clinical notes. Where there were discrepancies, the clinical note was considered more likely to represent what had occurred because therapists were more familiar with this form of documentation than the intervention CRF. Descriptive statistics were used to describe the quantity and content of the intervention delivered.

Intervention content was analysed by comparing each CRF with the clinical notes. The proportion of time spent on each intervention component was calculated from the CRF. Duplicated data were removed from the analysis and missing data were identified.

The frequency of VR intervention was calculated by identifying and summing each separate session. Intervention duration was calculated in months/weeks and days using the start and finish dates. Total time spent in direct contact and indirect contact with patients was taken from the intervention CRFs.

Descriptive statistics were used to describe the frequency, duration and mode of each monthly mentoring session across the four OTs. The results of this are reported elsewhere [25, 34].

Text describing factors moderating implementation fidelity (participant responsiveness, intervention complexity, facilitation strategies and quality of delivery) were extracted and then triangulated across multiple records (fidelity checklists, clinical notes, mentoring CRFs and interviews). Then, data were mapped to the CFIF moderating factors using an excel sheet.

Interview transcripts were analysed by at least two researchers using the framework method [36].

Ethical approval was granted by Integrated Research Approval System (REC Ref: 13/EM/0353 and the University of Nottingham Ethics Ref: D14112013 FRESH). The process for obtaining participant (patient participants, OT participants, employer participants, NHS staff participants) informed consent was in accordance with Research Ethics Committee guidance and Good Clinical Practice [37].

## Results

### Participants

Quantitative data from intervention CRFs were available for 38 people with TBI participating in the FRESH trial and randomised to receive the VR intervention. Using the Glasgow coma scale [38] to indicate TBI severity, approximately 50% (*n* = 19) had a mild TBI, a mean age of 40.4 years (range 16–62), 87% (*n* = 33) were male, and 71% (*n* = 27) were in full time work prior to injury.

Of the 38, 15 consented to interview, had a mean age of 39.4 years (range 25–61), 80% (*n* = 12) were male, six had a severe TBI, four a moderate TBI and five a mild TBI. Just over half (*n* = 7) were injured through falling, five from road traffic collisions, two from assaults and one was unsure. Six had other rehabilitation being delivered and five had occupational health services involved. Whilst all participants consented to the OT communicating with their employer, only seven consented to a workplace visit. Participants’ job roles included electrician, abattoir worker, carer, rigger, restaurant waiter, teacher, business owner, administrator, computing, warehouse worker, estates manager and doctor.

Five OTs (four women) were recruited with a mean age of 39.2 years (range 34 to 47 years). OTs were qualified a mean of 11.4 years (range 12 to 15 years). Two qualified in the UK and three overseas (South Africa, New Zealand and Australia). One held a higher degree in VR. All had experience in the NHS and with people with neurological conditions (mean 9.7 years, range 3–15 years). Two OTs worked for the NHS (community and acute), two were private practitioners. One OT left the trial. Two OTs were based in one site, the other two sites had one OT each.

Of the 15 TBI participants, 13 consented to their employer being contacted for interview, one was self-employed and one declined. Six employers consented and were interviewed. Four were line managers of the patient participant, one was a human resources manager and one an occupational health provider. They represented small, medium, and large employers. Two were third-sector organisations, two education facilities, one an NHS Trust and one a restaurant.

Thirteen NHS staff from four organisations (Community NHS Trusts, Acute NHS Trusts, NHS England, and a Clinical Commissioning Group) with varying roles (including research and development, strategic clinical network manager, commissioner, community occupational therapist, lead occupational therapist, clinical services manager, and a local clinical principal investigator) consented and were interviewed.

### Assessment of fidelity

Table 2 combines all the quantitative data sources (Intervention CRFs, clinical notes and fidelity checklists) and illustrates whether each OT delivered the intervention with fidelity according to the adherence constructs of CFIF (coverage, intervention content, duration and frequency) and indicates which type of moderating factor affected the delivery of the intervention. Overall, OTs delivered the FRESH VR with fidelity. The fidelity checklist indicated that the intervention was delivered as intended with core processes almost ‘always’ or ‘often’ followed by all therapists.

**Table 2:** Fidelity of FRESH intervention and identified moderating factors

Adherence			OTs				Moderating factors	Fidelity assessment
			OT A	OTB	OTC	OT D		
**Coverage**			✓	✓	✓	✓	Participant responsiveness, intervention complexity	Fidelity met in all cases
**Content**			✓	✓	✓	✓	Participant responsiveness, facilitation strategies, intervention complexity and context	Fidelity met in all cases
**Duration**			✓*^1^	✓*^1^	✓*^1^	✓*^1^	Participant responsiveness, intervention complexity and context	Fidelity met in most cases
**Frequency**	**1**	**≤ 10 days**	✓*^3^	✓*^1^	✓*^3^	**	Participant responsiveness, intervention complexity	Fidelity met in most cases
	**2**	**1–8 weeks**	✓	✓	✓	✓	Facilitation strategies and context	Fidelity met in all cases
	**3**	**≤ 8 weeks**	✓	✓	✓	✓	Intervention complexity and context	Fidelity met in all cases
	**4**	**4-8 weeks**	✓	✓	✓	✓	Intervention complexity and context	Fidelity met in all cases

✓—fidelity met; ✓*—fidelity met except for *n* = x cases; **—missing data; timepoint 1 within 10 days of referral; timepoint 2 OT contact every 1–2 weeks, case manager 6–8 weeks; timepoint 3 on graded RTW, weekly for 4 weeks, then fortnightly for 8 weeks, then checks ≤ 8 weeks; timepoint 4 on full RTW contact is 4–8 weeks; RTW—return to work

**Fig. 1:**
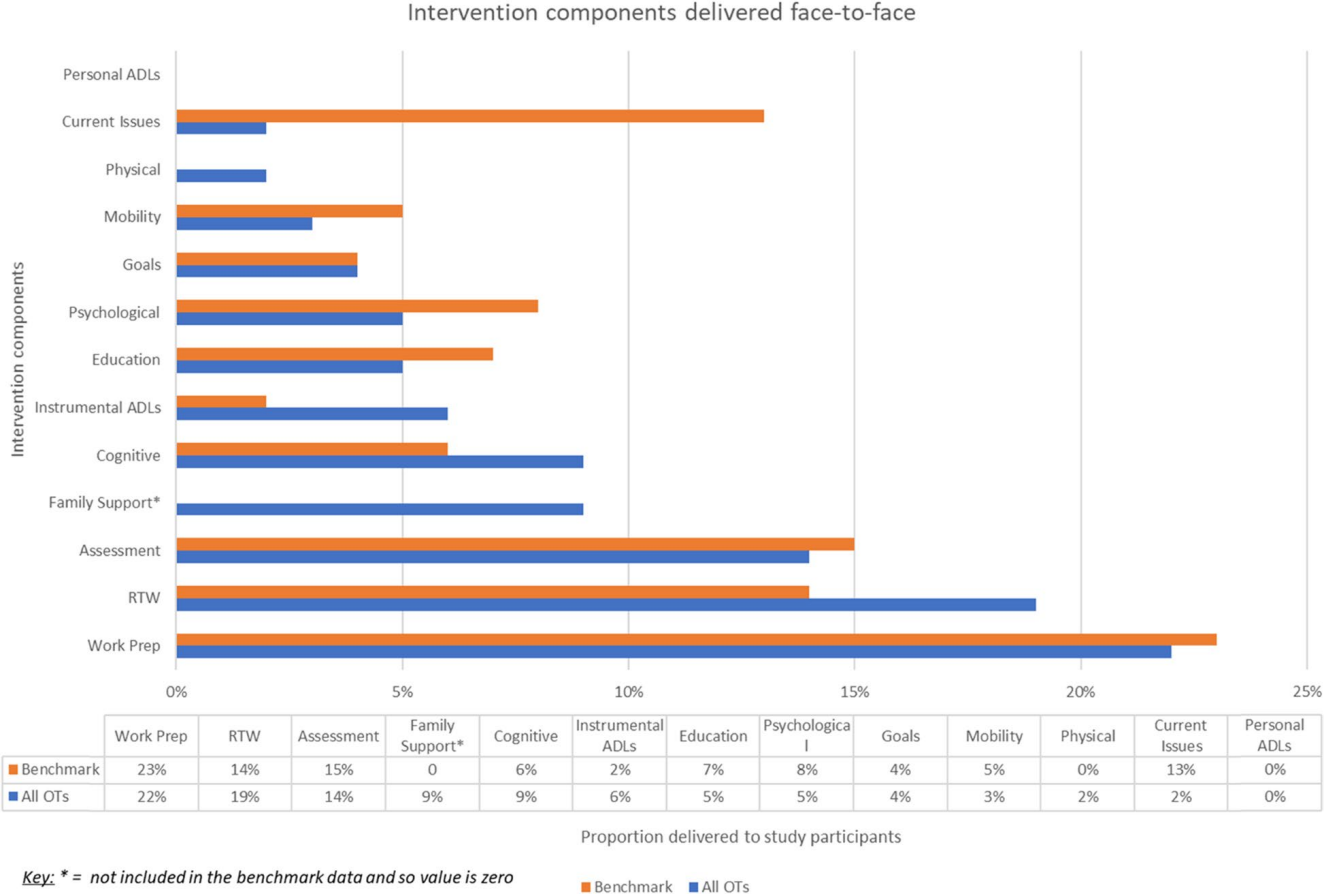
OT delivery of intervention components in comparison to benchmark

**Fig. 2:**
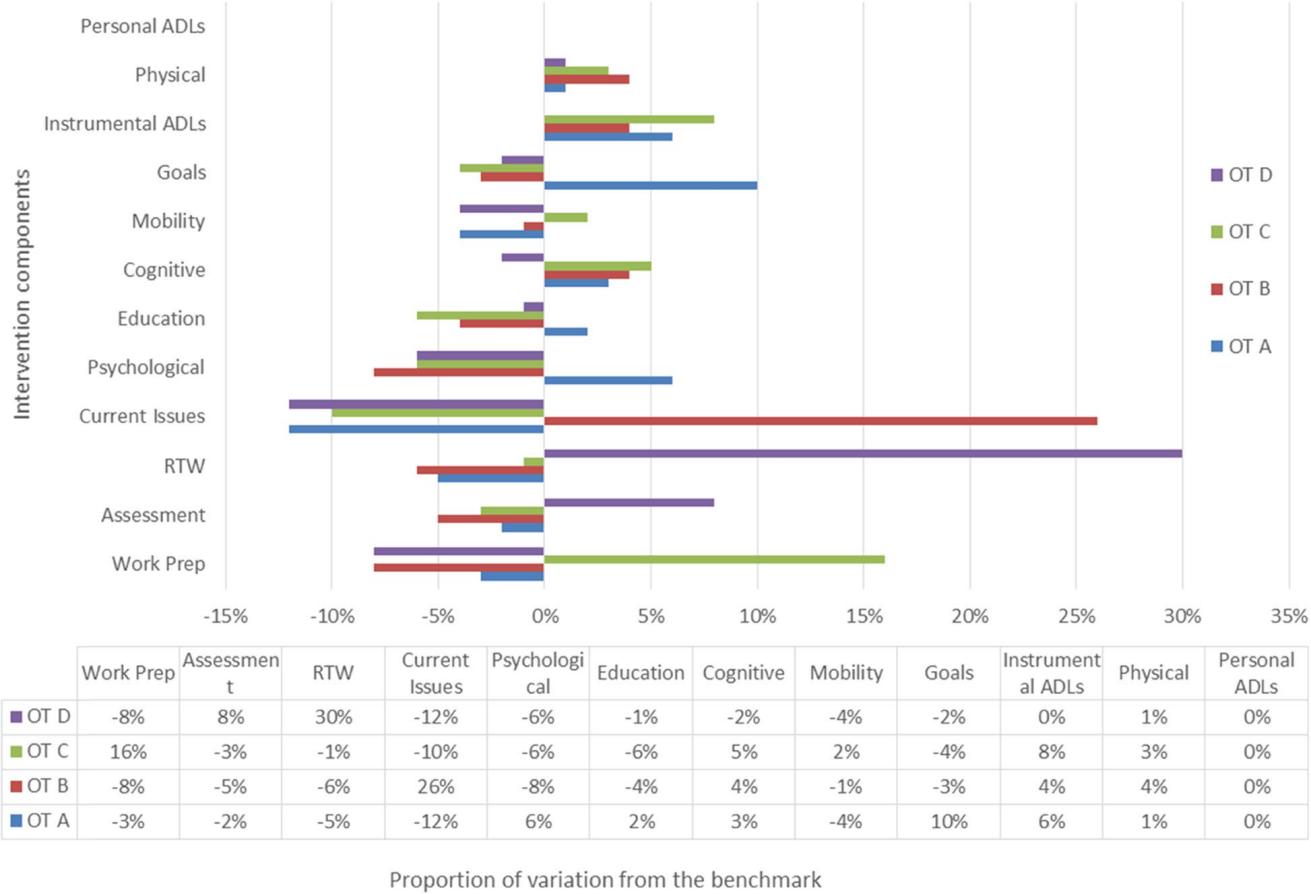
Individual OT variation in delivery of components compared to the benchmark

Figure 1 illustrates the mean proportion of time spent delivering each component by all OTs. Individual variations are shown in Fig. 2 where data are normalised with 0% representative of the benchmark.

Whilst most components were delivered with little (10%) variation, three components (current issues, RTW and work preparation) were delivered with greater variation. Moderating factors extracted from mentoring CRFs and clinical notes explained that this was due to tailoring the intervention to meet participants’ needs. For example, OT-D delivered more return to work due to a single participant who successfully returned to work but then experienced workplace relationship issues requiring additional OT support. OT-C delivered more work preparation because one participant had pre-existing addiction issues, and another had neuropsychological symptoms that required additional support before RTW. OT-B supported one participant extensively with current issues navigating multiple medical appointments.

Duration and frequency (dose) were recorded on the fidelity checklist as four key time points e.g. starts assessment within 10 days of recruitment. The results indicated close adherence to the key time points but with some variation. As expected, frequency of sessions was highest in month one and then declined. Based on the benchmark, it was anticipated that participants would receive approximately 11 sessions. The mean number of sessions across all participants and all OTs was 6.3 (range 0–40).

OTs demonstrated fidelity to the VR intervention duration, except each OT exceeded the 12-month duration with a single participant. Moderating factors explained reasons:

- OT-A had a participant who returned to the same job, same employer and remained on the OT’s caseload for 11 months but without receiving intervention and used the maximum 12-month allowable as a follow-up period in case any problems occurred (with job retention).
- OT-B had a participant who did not return to work and needed referrals to 10 further services to meet trauma-related needs. Lengthy NHS waiting times meant the therapist monitored the participant beyond 12 months, until these services were in place.
- OT-C had a participant who was not in regular work prior to recruitment. Intervention was recorded over 8 months and discharge was recorded close to 12 months. However, contact was made again at the 16-month point without clear reasons.
- OT-D had a participant who returned to studying. Clinical records provided no clear reason for discharging the participant after the 12-month point.

### Feasibility findings

#### Feasibility of data collection

A large amount of data was collected all of which required the individual attention of researchers to administer their collection, data cleansing and storage prior to analysis. Thirty-eight sets (one per trial participant) of hard copy clinical notes, 699 (42–248 per therapist) intervention CRFs and 12 fidelity checklists (one per OT over three visits) were obtained. Qualitative data was extracted from all clinical notes, the size of which ranged from approximately 10 to over 50 pages, 12 fidelity checklists, 183 mentoring CRFs and 38 interview transcripts. Interviews ranged from approximately 30–90 min, which reflected the length of transcripts.

We had intended to collect more data. For example, it was planned that 16 fidelity checklists would be collected but due to OTs workloads it was not possible to schedule the planned four visits per OT. We had also hoped to interview more employers, but this group proved hardest to reach. Employers were not always willing to agree to the time required for interview (approximately 30 min), or they did not feel they anything worthwhile to offer or even after consenting did not attend the telephone interview. In terms of NHS staff, individual commissioners of TBI-related rehabilitation were the most difficult and took the most time to identify.

CRFs were mostly completed as directed. OTs organised their clinical notes according to local practices and this resulted in a lack of uniformity, which increased time required to locate information. Handwritten clinical notes were not always legible and typed notes were quickest to read.

The fidelity checklist followed a previously published example found to be implementable [23]. The list of items were based on a robust description of the ESTVR intervention using the template for intervention description and replication (TIDieR) guide [39], making it straightforward to use. However, arranging and conducting fidelity assessment visits with the OTs, plus travel and associated costs made this method resource intensive.

The data most feasible to collect were mentoring records as these were written and stored by mentors who were part of the research team. All other data collection methods were resources intensive, with interviewing employers and commissioners proving to be most challenging.

Table 3 summarises how feasible it was to collect and analyse the fidelity data and identify factors affecting fidelity. Recommended methods are identified as are those that were considered resource intensive and methods that provided least added value to the fidelity assessment.

**Table 3:** Summary of the feasibility of fidelity measurement methods

	Recommended tools and methods	Resource intense methods	Methods that added least value
**Data collection**	Mentoring CRF Intervention CRF Clinical notes	Arranging interviews Fidelity visits	Interviews
**Data analysis**	Triangulation of intervention delivery records Comparison with a benchmark	Triangulation Interview analysis	Interview analysis
**Fidelity assessment**	Fidelity checklist Intervention CRF Clinical notes	Fidelity visits	Interviews
**Identifying factors affecting fidelity**	Clinical notes Mentor CRFs Mapping against CFIF framework	Interview analysis Reviewing clinical notes	Interviews

#### Feasibility of data analysis

Triangulation of data sources successfully highlighted missing data between clinical notes and CRFs, for example, OTs did not always record time spent in letter writing on the intervention CRF and intervention sessions recorded on the CRFs were not routinely documented in clinical notes. Although triangulation was straightforward, the time required to manually search and compare CRFs and clinical notes was considerable.

Comparing the fidelity results to a benchmark was quick and straightforward. However, the adaptation of the intervention CRF from the original study by Phillips [33] meant it was not possible to compare the ‘family support’ component with the benchmark.

Mapping relevant portions of text extracted from clinical notes, fidelity checklists, mentoring CRFs and interviews to the CFIF moderating factors constructs highlighted and explained what affected intervention delivery. Whilst interviewing the participants and OTs sometimes helped to clarify these factors, the interview data from NHS staff and employers did not add new insights for fidelity. All interviews did, however, provide important contextual detail related to broader implementation of VR and useful for future TBI rehabilitation research.

Exploring the moderating factors revealed where changes needed to occur to improve fidelity in the future. For example, changes to the intervention manual and descriptions of the intervention to provide greater clarity around intervention duration and discharge procedures.

All data analysis methods required extensive researcher resources. Triangulation was essential to plug the gaps left when OTs did not consistently complete CRFs or document intervention delivery in clinical notes. The use of a framework helped in organising and interpreting the large volume of data.

#### Feasibility of identifying moderating factors

Data collection tools that described ‘why’ OTs did not deliver certain intervention components or did not follow processes proved most revealing in terms of understanding fidelity. These included clinical notes, mentor records and fidelity visits with OTs. Interviews were helpful for revealing the broader contextual issues that affected implementation, but they did add value to the fidelity assessment.

## Discussion

This study designed a new method to assess fidelity of a complex VR intervention delivered by OTs in the FRESH feasibility RCT. The theoretical framework of CFIF provided the overall structure and the multiple data sources and mixed methods revealed that OTs delivered the VR to TBI participants with fidelity. This method enabled a detailed and rigorous analysis of fidelity that also helped identify factors that moderated intervention delivery and explain fidelity violations (deviations in process or component delivery). The fidelity assessment in this study was situated in the process evaluation. Doing so allowed data sources to be used for different purposes and reduced the burden on participants, for instance, interviews with OTs were used to evaluate the training package as well as exploring barriers and facilitators to delivering the VR intervention in FRESH. Assessing whether interventions are delivered as planned helps trialists understand effectiveness outcomes [12] and researchers to understand implementation issues for future trials [40]. This learning has since informed fidelity assessment in other trials [26, 41].

This study has indicated the most useful methods for measuring fidelity in a future trial, so that we better understand why a complex intervention works or fails [42] and improve future trial designs [43]. The data collection tools addressed different fidelity questions. We determined the optimum tools to measure fidelity were intervention CRFs, clinical notes and the fidelity checklist to measure what and how much intervention was delivered. Descriptions of intervention delivery in clinical notes, fidelity checklists and mentoring CRFs indicated whether intervention processes were followed and along with some interviews, explained moderating factors. However, the intensity of data collection and the need for greater contextual understanding of the trial findings should be balanced with collecting only what is necessary for investigating the effectiveness of the intervention [27].

Whilst multiple data sources corroborated findings and facilitated interpretation of moderating factors from different perspectives, there was redundancy in the qualitative measures of fidelity. For example, interviews with TBI participants indicated issues (moderating factors) relating to how needs changed over time, which were also documented in the OT’s clinical notes. Trial OTs’ frustrations in communicating with participants were reported in both OT interviews and mentoring CRFs. Given the resource implications of conducting, transcribing and analysing interviews, it could be argued that mentoring records alone may be more practical in a future trial [27]. Further investigations could test the adaptation of an existing patient and service provider-facing reporting form [29] for use with third parties such as employers and NHS staff to replace interviews.

The optimum methods of assessing fidelity were triangulation and comparison against a benchmark. Triangulation revealed useful information about trial-related issues that were broader than fidelity, for example missing data, the OTs’ training needs which is consistent with others’ findings, and offered insight into the intervention complexity, and the measurement processes. These useful insights provided the opportunity to reflect on learning going forward. Especially, pertinent was the issue of missing data and the need to address accurate and consistent CRF completion in future training in complex intervention trials. Triangulation was resource intensive and involved large volumes of data that required hours of analysis. In a future study, this could be reduced by sampling a proportion of participant data, for instance, randomly selecting a participant per site as a case study or sampling 5–10% of therapists’ caseload. Sampling methods could also consider the amount of experience therapists might gain over time or by the number of participants seen. Only using efficient measurement tools (fidelity checklist, intervention CRFs and mentoring CRFs is also recommended.

The benchmark, which was derived from the description of the VR intervention delivered in an earlier study (33), offered quality assurance that ESTVR was delivered with fidelity despite anticipated variation in delivery. Variation was expected because of complexities associated with TBI, the intervention and work context and the fact that the VR was delivered by different OTs in different sites. Whilst some variation is concerned with improving the fit of the intervention [44, 45], it may also be seen as non-adherence, negatively impacting on patient outcomes [46].

Understanding variations in intervention implementation during trials and potential effects on patient outcomes is important [47]. Using Stirman’s (2013) system of coding variations to intervention delivery, the most frequently type observed in this study was ‘tailoring’. Analysis of fidelity at an individual level provides insights and opportunities to learn that group-level data cannot. For example, our data demonstrated that a single therapist’s delivery of an intervention can skew the overall picture of what was delivered to research participants. We therefore explored both variation across and between OTs against the benchmark in terms of content and dose, which provided greater clarity about how OTs delivered VR. Trialists should examine this variation to understand implementation in real-world contexts and minimise dilution effects whilst achieving appropriate adaptation for local contexts [48]. In this study, the range of fidelity measurement tools provided reassurance that variation was not nonadherence.

Some agreement is required as to what is an acceptable level of variation. The findings in this study suggest that variation of up to 15% in intervention component delivery across all therapists may be acceptable but variation greater than this should be explored. Tailoring a complex intervention may result in variation and qualitative data should be able to explain this. Providing it remains below 40%, this remains acceptable and consistent with others [14].

The fidelity checklist provided a useful insight into fidelity at different time points, something Masterson-Algar (2014) recognised as important, as therapists gain more experience [18]. However, it did not adequately reflect ‘tailoring’ for example, when it was inappropriate to deliver a component, or if a timeframe could not be met. Further refinement of the checklist is required to include a scoring system that takes account of intervention tailoring. Since we initially developed this checklist, others have developed similar tools. Walton used a checklist to measure fidelity in a community occupational therapy intervention for people with dementia [49]. Our checklist has been developed incorporating newer literature, such as Walton’s work and used in another VR trial for stroke survivors [26].

Face-to-face fidelity monitoring visits were time consuming, but valuable [50]. The researcher (JP) who assumed the dual role of trainer and mentor was an experienced OT and academic who enabled a professional relationship with the trial OTs. This permitted in-depth enquiry about fidelity, which might not have been possible with someone less experienced. Whilst in future trials fidelity monitoring could be done by clinical-academic mentors, given the resource implications, it seems important to determine whether non-specialists could use a fidelity checklist alongside detailed guidance notes.

On occasions, the OTs struggled to deliver the intervention with fidelity; this was revealed in real-time through mentoring as well as recorded on the CRFs. Mentoring helped to prevent ‘drift’ from the core process and the risk of shift towards a different intervention. This approach allowed beneficial adaptations to be recorded, which is important in intervention development and to inform future implementation in other settings and a future phase III trial [27].

Although these findings report fidelity assessment of only four OTs who implemented a new complex intervention and are unlikely to be representative of all therapists, they highlight important points for consideration when training OTs to deliver complex interventions and measuring fidelity in a trial context.

## Conclusions

OTs delivered the VR intervention with fidelity but also with variation, as expected, and this was measured by data from multiple sources. This was useful in a feasibility trial because it identified factors likely to affect intervention fidelity in the future. However, multiple methods answer different questions. Fidelity checklists answer whether intervention processes were followed and explain the moderators. Adherence is answered with intervention CRFs and clinical notes. Only expert mentoring provides real-time indicators to fidelity deviations and why. Focussing resources on providing mentoring to therapists delivering an intervention should be considered an important facilitatory tool for implementation fidelity that affords multiple benefits. Some methods do not add value to fidelity measurement and may be wasteful of resources. Qualitative interviews with OTs, participants, employers and NHS staff did not provide additional useful data for fidelity measurement. Unanswered questions remain regarding non-specialists measuring fidelity and what is an acceptable variation of fidelity.

## Supplementary Information

**Additional file 1.**

**Additional file 2.**

**Additional file 3.**

**Additional file 4.**

**Additional file 5.**

**Additional file 6.**

## Data Availability

The datasets used and/or analysed during the current study are available from the corresponding author on reasonable request.

## Abbreviations

CFIF: The Conceptual Framework for Implementation Fidelity
CRF: Case report form
DWP: Department for Work and Pensions
FRESH: Facilitating Return to work through Early Specialist Health-based interventions: feasibility randomised controlled trial
GCS: Glasgow coma scale
GP: General practitioner
HCPC: Health and Care Professions Council
HTA: Health Technology Assessment
NHS: National Health Service
NIHR: National Institute for Health Research
OH: Occupational health
OT: Occupational therapist
PwTBI: Person with a traumatic brain injury
REC: Research ethics committee
RCT: Randomised controlled trial
RTW: Return(ing) to work
TBI: Traumatic brain injury
VR: Vocational rehabilitation

## References

[1] Dewan MC, Rattani A, Gupta S, Baticulon RE, Hung Y-C, Punchak M (2019). Estimating the global incidence of traumatic brain injury. J Neurosurgery JNS..

[2] Headway. Traumatic brain injury: Headway; 2020 [Available from: https://www.headway.org.uk/about-brain-injury/individuals/types-of-brain-injury/traumatic-brain-injury/.

[3] Parsonage M. Traumatic brain injury and offending: an economic analysis [Online]. Available at: https://www.centreformentalhealth.org.uk/traumatic-brain-injury [Accessed 29 May 2017]: Centre for Mental Health; 2016.

[4] Simpson G, Tate R (2007). Suicidality in people surviving a traumatic brain injury: prevalence, risk factors and implications for clinical management. Brain Injury..

[5] Cancelliere C, Kristman VL, Cassidy JD, Hincapie CA, Cote P, Boyle E (2014). Systematic review of return to work after mild traumatic brain injury: results of the international collaboration on mild traumatic brain injury prognosis. Arch Phys Med Rehabil..

[6] Materne M, Lundqvist LO, Strandberg T (2017). Opportunities and barriers for successful return to work after acquired brain injury: A patient perspective. Work..

[7] van Velzen JM, van Bennekom CAM, Edelaar MJA, Sluiter JK, Frings-Dresen MHW (2009). How many people return to work after acquired brain injury?: A systematic review. Brain Injury..

[8] Playford E, Radford K, Burton C, Gibson A, Jellie B, Sweetland J (2011). Mapping vocational rehabilitation services for people with long term neurological conditions: summary report.

[9] Saltychev M, Eskola M, Tenovuo O, Laimi K (2013). Return to work after traumatic brain injury: systematic review. Brain injury.

[10] Escorpizo R, Finger M, Glässel A, Cieza A (2011). An international expert survey on functioning in vocational rehabilitation using the international classification of functioning, disability and health. J Occup Rehabil..

[11] Skivington K, Matthews L, Simpson SA, Craig P, Baird J, Blazeby JM (2021). A new framework for developing and evaluating complex interventions: update of Medical Research Council guidance. Bmj..

[12] Dusenbury L, Brannigan R, Falco M, Hansen WB (2003). A review of research on fidelity of implementation: implications for drug abuse prevention in school settings. Health Education Res..

[13] Cucciare MA, Curran GM, Craske MG, Abraham T, McCarthur MB, Marchant-Miros K, et al. Assessing fidelity of cognitive behavioral therapy in rural VA clinics: design of a randomized implementation effectiveness (hybrid type III) trial. Implementation Sci. 2016;11(1):1-9. 10.1186/s13012-016-0432-4 PMC4862056 27164866

[14] Durlak JA, DuPre EP (2008). Implementation matters: a review of research on the influence of implementation on program outcomes and the factors affecting implementation. Am J Community Psychol..

[15] Forgatch M, Patterson G, DeGarmo D (2005). Evaluating fidelity: predictive validity for a measure of competent adherence to the oregon model of parent management training. Behav Ther..

[16] Bowyer P, Tkach MM (2018). Treatment fidelity in Model of Human Occupation research. Brit J Occup Ther..

[17] Hand BN, Darragh AR, Persch AC (2018). Thoroughness and psychometrics of fidelity measures in occupational and physical therapy: a systematic review. Am J Occup Ther..

[18] Masterson-Algar P, Burton CR, Rycroft-Malone J, Sackley CM, Walker MF (2014). Towards a programme theory for fidelity in the evaluation of complex interventions. J Eval Clin Pract..

[19] Carroll C, Patterson M, Wood S, Booth A, Rick J, Balain S (2007). A conceptual framework for implementation fidelity. Implement Sci.

[20] Hildebrand MW, Host HH, Binder EF, Carpenter B, Freedland KE, Morrow-Howell N (2012). Measuring treatment fidelity in a rehabilitation intervention study. Am J Phys Med Rehabil.

[21] Bellg A, Borrelli B, Resnick B, Hecht J, Minicucci D, Ory M, Ogedegbe G, Orwig D, Ernst D, Czajkowski S (2004). Enhancing treatment fidelity in health behaviour change studies: best practices and recommendations from the NIH Behavior Change Consortium. Health Psychol.

[22] Breitenstein SM, Gross D, Garvey C, Hill C, Fogg L, Resnick B (2010). Implementation fidelity in community-based interventions. Res Nursing Health..

[23] Hasson H, Blomberg S, Dunér A (2012). Fidelity and moderating factors in complex interventions: a case study of a continuum of care program for frail elderly people in health and social care. Implement Sci.

[24] Holmes JA, Logan P, Morris R, Radford K (2020). Factors affecting the delivery of complex rehabilitation interventions in research with neurologically impaired adults: a systematic review. Syst Rev.

[25] Radford K, Sutton C, Sach T, Holmes J, Watkins C, Forshaw D (2018). Early, specialist vocational rehabilitation to facilitate return to work after traumatic brain injury: the FRESH feasibility RCT. Health Technol Assess..

[26] Radford KA, McKevitt C, Clarke S, Powers K, Phillips J, Craven K (2022). RETurn to work After stroKE (RETAKE) Trial: protocol for a mixed-methods process evaluation using normalisation process theory. BMJ open..

[27] Toomey E, Hardeman W, Hankonen N, Byrne M, McSharry J, Matvienko-Sikar K (2020). Focusing on fidelity: narrative review and recommendations for improving intervention fidelity within trials of health behaviour change interventions. Health Psychol Behav Med.

[28] Toomey E, Matthews J, Hurley DA (2017). Using mixed methods to assess fidelity of delivery and its influencing factors in a complex self-management intervention for people with osteoarthritis and low back pain. BMJ Open..

[29] Walton H, Spector A, Williamson M, Tombor I, Michie S (2020). Developing quality fidelity and engagement measures for complex health interventions. Brit J Health Psychol.

[30] Siemonsma P, Dopp C, Alpay L, Tak E, van Meeteren N, Chorus A (2014). Determinants influencing the implementation of home-based stroke rehabilitation: a systematic review. Disabil Rehabil..

[31] Radford KA, Phillips J, Jones T, Gibson A, Sutton C, Watkins C, et al. Facilitating return to work through early specialist health-based interventions (FRESH): protocol for a feasibility randomised controlled trial. Pilot Feasibility Studies. 2015;1(24). 10.1186/s40814-015-0017-z PMC5154052 27965803

[32] Holmes J, Phillips J, Morris R, Bedekar Y, Tyerman R, Radford K (2016). Development and evaluation of an early specialised traumatic brain injury vocational rehabilitation training package. Brit J Occup Ther..

[33] Phillips J, Drummond A, Radford K, Tyerman A (2010). Return to work after traumatic brain injury: recording, measuring and describing occupational therapy intervention. Br J Occup Ther..

[34] Holmes JA. Implementing complex rehabilitation interventions in research: the example of vocational rehabilitation for people with traumatic brain injury: University of Nottingham; 2018.

[35] Phillips J. Return to work after traumatic brain injury: a cohort comparison study and feasibility economic analysis: University of Nottingham; 2013. 10.3109/02699052.2013.766929 23473058

[36] Gale NK, Heath G, Cameron E, Rashid S, Redwood S (2013). Using the framework method for the analysis of qualitative data in multi-disciplinary health research. BMC Med Res Methodol.

[37] Vadivale M. ICH-GCP guidelines for clinical trials. Berita MMA. 1999;7(29).

[38] Teasdale G, Maas A, Lecky F, Manley G, Stocchetti N, Murray G (2014). The Glasgow Coma Scale at 40 years: standing the test of time. Lancet Neurol.

[39] Hoffmann TC, Glasziou PP, Boutron I, Milne R, Perera R, Moher D (2014). Better reporting of interventions: template for intervention description and replication (TIDieR) checklist and guide. BMJ.

[40] Poltawski L, Norris M, Fau - Dean S, Dean S. (2014). Intervention fidelity: developing an experience-based model for rehabilitation research. J Rehabil Med.

[41] Kendrick D, das Nair R, Kellezi B, Morriss R, Kettlewell J, Holmes J (2021). Vocational rehabilitation to enhance return to work after trauma (ROWTATE): protocol for a non-randomised single-arm mixed-methods feasibility study. Pilot and Feasibility. Studies..

[42] Walker MF, Hoffmann TC, Brady MC, Dean CM, Eng JJ, Farrin AJ (2017). Improving the development, monitoring and reporting of stroke rehabilitation research: consensus-based core recommendations from the Stroke Recovery and Rehabilitation Roundtable. Int J Stroke..

[43] Allen JD, Linnan LA, Emmons KM. Fidelity and its relationship to implementation effectiveness, adaptation, and dissemination. In: Brownson RC, Colditz GA, Proctor EK, editors. Dissemination and implementation research in health: translating science to practice: Oxford University Press; 2012.

[44] Hawe P, Shiell A, Riley T, Gold L (2004). Methods for exploring implementation variation and local context within a cluster randomised community intervention trial. J Epidemiol Community Health..

[45] Moore GF, Audrey S, Barker M (2015). Process evaluation of complex interventions: Medical Research Council guidance. Bmj..

[46] Mihalic S (2004). The importance of implementation fidelity. Emotional Behav Disord Youth..

[47] Stirman SW, Miller CJ, Toder K, Calloway A (2013). Development of a framework and coding system for modifications and adaptations of evidence-based interventions. Implement Sci..

[48] McCrabb S, Lane C, Hall A, Milat A, Bauman A, Sutherland R (2019). Scaling-up evidence-based obesity interventions: a systematic review assessing intervention adaptations and effectiveness and quantifying the scale-up penalty. Obesity Rev.

[49] Walton H, Tombor I, Burgess J, Groarke H, Swinson T, Wenborn J (2019). Measuring fidelity of delivery of the Community Occupational Therapy in Dementia-UK intervention. BMC Geriatr.

[50] Chesworth BM, Leathley MJ, Thomas LH, Sutton CJ, Forshaw D, Watkins CL (2015). Assessing fidelity to treatment delivery in the ICONS (Identifying Continence OptioNs after Stroke) cluster randomised feasibility trial. BMC Med Res Methodol.

