# Supplementary material for: Developing a method to assess fidelity to a complex vocational rehabilitation intervention in the FRESH trial: a feasibility study
Source: Pilot Feasibility Stud. 2022 Jul 29;8:160. doi: 10.1186/s40814-022-01111-2 (PMC9335967; doi:10.1186/s40814-022-01111-2)
Supplement: Supplementary file 1 — Additional file 1. [file 40814_2022_1111_MOESM1_ESM.docx]

Additional File 1: Flow diagram – Facilitating Return to work through Early Specialist Health-based interventions (FRESH) study Process evaluation

**To describe participating sites (Months 1-23)**

- Site feasibility questionnaire
- Telephone survey

**Development of FRESH Intervention Training Package** (manual, training and mentoring)

**(Months 1-6)**

Therapist Pre-training knowledge & confidence questionnaire

**To understand OTs’ experiences of being trained and delivering intervention**

**(Months 12-14 & 32-34)**

- Interviews with FRESH OTs.

**Therapist Training**

**(Months 7-9)**

Therapist Post-training

Knowledge & confidence questionnaire

**To describe dose, content, adherence and barriers to delivery (implementation):**

- Intervention CRF (session content) (Month 34-38)
- NHS therapy notes (Months 13-38)
- Fidelity checklists at 3-monthly monitoring visits (Months 13-38)
- Mentoring records (Monthly)
- Interviews with participants, therapists, employers & NHS staff (Months 15-34)

**FRESH Intervention Delivery in 3 Sites (Months 9-34)**

Manual-based FRESH delivered by a trained OT supported by monthly expert and peer mentoring

**Refresher Training** (sites 1-3)

**(Month 13)**

**Usual Care Only**

**To describe content and delivery of Usual Care:**

- Site level questionnaire
- Self-reported resource use data.
- Interviews with participants (n=15, 5 in each site)

**To explore participants experiences of taking part in the intervention and perceptions and experiences of support to return to work (Months 22-34)**

- Interviews with participants receiving VR and Usual Care (n=30, 5 in each site)

**Understand the social and structural factors which support the implementation of the intervention (Months 16-38)**

- Face to face semi-structured interviews with FRESH OT delivering the FRESH intervention at each site at the end of the study
- De-brief meetings within sites at the end of the trial

**Synthesis of Process Evaluation Data with Trial outcome data (Months 12-38)**

OT – occupational therapist
