# Supplementary material for: Developing a method to assess fidelity to a complex vocational rehabilitation intervention in the FRESH trial: a feasibility study
Source: Pilot Feasibility Stud. 2022 Jul 29;8:160. doi: 10.1186/s40814-022-01111-2 (PMC9335967; doi:10.1186/s40814-022-01111-2)
Supplement: Supplementary file 2 — Additional file 2. [file 40814_2022_1111_MOESM2_ESM.docx]

Additional file 2: The logic model of the Facilitating Return to work through Early Specialist Health-based interventions (FRESH) vocational rehabilitation (VR) intervention

| **Resources** | **Core process inputs in-puts from OT** | **Short term Impacts** | **Impacts** | **Health Outcomes** |
| --- | --- | --- | --- | --- |
| Facilitating legal framework & policies  Skilled, knowledgeable TBI/VR therapist & patient with a job  Co-location - crossing boundaries between health, employment, charities  Multi-stakeholder engagement | Intervenes within 4 weeks of injury, advises on impact of injury & RTW.  Provides ongoing education, advice & emotional support to patient and family  Coordinates patient’s vocational rehabilitation across all sectors  Communicates openly in writing with stakeholders re: work performance.  Assesses impact of TBI on person & job, analyses impact on work ability.  Delivers individually tailored vocational rehabilitation in the community.  Explores alternatives to pre-injury employment when existing employment not feasible or sustainable.  Adapts workplace, negotiates phased RTW, provides feedback on performance  Monitors RTW to ensure work stability | Patient does not make rapid RTW decisions.  Patient aware of available support & how to access.  Vocational Case coordinator has early and regular contact with the trauma survivor, family, and other stakeholders e.g. employer, GP, DWP, OH  Patient & stakeholders aware of residual problems & coping strategies  Patient, employer aware of impact of TBI on work.  Patient & stakeholders aware of residual TBI problems.  Coping strategies in place  Referrals made for relevant support | Patient & employer satisfaction.  Patient confident & able to self-manage  Stakeholders report to one key contact, all aware of stakeholders involved and work towards RTW  Conflicting RTW advice prevented  Everyone is heard, no confusing communications  Considered decisions made re RTW  Patient & employer satisfaction  Employment environment Optimised  Workplace accommodations in place  Phased return to work facilitated  Patient able to cope with work  Contributes to economy | Prevent job loss  Improved physical and mental health  Personal and financial wellbeing  Reduced sickness absence  Reduced health resource use  Patient in sustainable employment |

*Key: OT – occupational therapist, TBI – traumatic brain injury, VR – vocational rehabilitation, RTW = return to work, GP = General Practitioner (Physician), DWP = Department for Work and Pensions, OH = Occupational Health*
