# Supplementary material for: Developing a method to assess fidelity to a complex vocational rehabilitation intervention in the FRESH trial: a feasibility study
Source: Pilot Feasibility Stud. 2022 Jul 29;8:160. doi: 10.1186/s40814-022-01111-2 (PMC9335967; doi:10.1186/s40814-022-01111-2)
Supplement: Supplementary file 3 — Additional file 3. [file 40814_2022_1111_MOESM3_ESM.docx]

**Additional File 3: Description Facilitating Return to work through Early Specialist Health-based interventions (FRESH) Vocational Rehabilitation Intervention using The TIDieR (Template for Intervention Description and Replication) Checklist**

**Intervention name** - FRESH Vocational Rehabilitation (VR) Intervention

**Rationale of the intervention**

FRESH VR was an early traumatic brain injury (TBI) specialist vocational rehabilitation intervention received by patients in the ‘Facilitating return to work through early specialist health-based interventions’ (FRESH) trial. The full FRESH clinical trial protocol is available via the NIHR website: http://www.nets.nihr.ac.uk/projects/hta/116602.

The intervention developed from an early TBI specialist VR intervention delivered in the NHS by an Occupational Therapist (OT), supported by a TBI Case Manager. This novel National Health Service (NHS) intervention was compared to usual NHS rehabilitation in a single centre cohort comparison involving 94 patients with TBI of all severities. The findings suggested better work outcomes may be achieved through early occupational therapy targeted at job retention (1).

FRESH VR aimed to prevent job loss in people who were employed or in fulltime education prior to their TBI. FRESH VR used a case coordination model that involved identifying employed people within eight weeks of injury, assessing the impact of the TBI injuries on the person and work/education activities, roles and responsibilities. The assessment identified and addressed problems that were related to return to work/education. Individualised intervention then focussed on developing acceptable ways of overcoming problems in conjunction with the employer/education provider.

**Resources required to deliver FRESH VR**

The intervention was delivered and monitored by qualified OTs with experience in acquired brain injury and registered with the Health and Care Professionals Council and (ideally) employed by NHS trusts. Participating occupational therapists were trained to deliver the intervention in their own NHS Trust. OTs delivered all of the face-to-face contacts with patients. Training involved a hardcopy manual of the intervention together with two-days of direct instruction from a training team comprised of OTs with expertise in VR and acquired brain injury and patient representatives plus additional invited speakers related to case management. Six months later a follow up refresher day was provided. All OTs received individual hourly monthly sessions for the entire duration of the FRESH trial with a mentor who was part of the training team. Further details of the training is available (2, 3).

The training materials provided resources related to TBI and return to work/study that were readily available online to use with patients and employers.

**Procedures, when and how much**

The intervention was multifaceted and must be tailored and involved these core activities.

1. **Assessment**

Minimum standards are that patients will receive initial and ongoing assessment where the OT received referral within 8-weeks, typically at 5-weeks post TBI, made contact regardless of where patient is located within 10 working days of referral and used a range of assessment tools that address rehabilitation and VR needs. The OT then assessed the impact of TBI on function and work/study role, assessed the work/study role, work duties/functions, work/job demands and developed specific goals and a vocational rehabilitation plan. A risk assessment of the home environment to enable safe home/community visits was conducted.

1. **Early education and advice**

At minimum, patients received TBI & RTW education and support patient & family with specific advice to delay decisions about RTW until impact of TBI is understood and coping strategies formulated. Ongoing individualised education, advice, emotional support to patient, family was provided by the OT throughout VR process to ensure ongoing sessions with the OT every 1-2 weeks.

1. **Coordination and communication**

At minimum, patients were provided with a coordinated programme where the OT established which other health and community services were involved, collected relevant information about the organisation to coordinate vocational rehabilitation. The OT ensured that non-work/study focused activities remained on-going, actively liaised and communicated in writing with everyone involved, actively communicated with the employer, where permitted, about rehabilitation goals and supported the patient before and after work meetings. The OT planned in consultation with the patient and informed all parties about discharge from the intervention.

1. **Flexible, individually tailored preparation to RTW**

At minimum, patients had a flexible individually tailored programme where RTW options were explored with all parties including recommendations for any adaptations to work tasks, tools and environment. Work/study skills/functions were practiced, or retraining was organised and strategies were developed with the patient to communicate the impact of their TBI to colleagues and others. Coping strategies were developed with the patient to manage effects of their TBI in everyday life and work/study and alternatives to pre-injury employment/education were explored in cases where return to pre-existing employer is not feasible or is unsustainable.

1. **Supported and graded RTW**

At minimum, patients were supported to negotiate graded RTW for reintegration where the OT provided monitoring for safety and sustainability over the RTW period, every 2-5 days early on reducing to every 2 months later. The patient was supported in seeking and accepting feedback about their (work/study) function and employer supported to provide feedback. Coping skills were reinforced to deal with job retention risks.

1. **Discharge from the intervention**

FRESH VR lasted a maximum of 52 weeks with one or two contacts per month, but the frequency was variable according to the tailoring of the intervention. Early sessions took place in inpatient facilities, often a specialist trauma or rehabilitation unit. Many people were discharged from inpatient care after the first few weeks when intervention continued in the community, including the patient’s home or workplace or another agreed community venue

**Tailoring of the intervention**

FRESH VR was designed to be tailored from the point of assessment and continues throughout the core activities 1-6. Tailoring was based on taking into account patient goals, the complex national and local systems of the health service, Government welfare, TBI-related charities plus the individual differences of how each participant with traumatic brain injury (PwTBI) presents, their social situations and the interactions with every person’s job/study course and workplace/educational provider. The point at which discharge was made was also tailored to optimise the success of maintaining employment/education retention

Whether an OT directly engaged with the employer/education provider was tailored according to patient consent.

**References**

1. Radford K, Phillips J, Drummond A, Sach T, Walker M, Tyerman A, et al. Return to work after traumatic brain injury: cohort comparison and economic evaluation. Brain Injury. 2013;27(5):507-20.

2. Holmes J, Phillips J, Morris R, Bedekar Y, Tyerman R, Radford K. Development and evaluation of an early specialised traumatic brain injury vocational rehabilitation training package. British Journal of Occupational Therapy. 2016;79(11):693-702.

3. Holmes JA. Implementing complex rehabilitation interventions in research: the example of vocational rehabilitation for people with traumatic brain injury: University of Nottingham; 2018.
