# Supplementary material for: Developing a method to assess fidelity to a complex vocational rehabilitation intervention in the FRESH trial: a feasibility study
Source: Pilot Feasibility Stud. 2022 Jul 29;8:160. doi: 10.1186/s40814-022-01111-2 (PMC9335967; doi:10.1186/s40814-022-01111-2)
Supplement: Supplementary file 4 — Additional file 4. [file 40814_2022_1111_MOESM4_ESM.docx]

## Additional File 4: Intervention session content CRF
