# Supplementary material for: Developing a method to assess fidelity to a complex vocational rehabilitation intervention in the FRESH trial: a feasibility study
Source: Pilot Feasibility Stud. 2022 Jul 29;8:160. doi: 10.1186/s40814-022-01111-2 (PMC9335967; doi:10.1186/s40814-022-01111-2)
Supplement: Supplementary file 5 — Additional file 5. [file 40814_2022_1111_MOESM5_ESM.docx]

**Additional File 5: Fidelity Visit Checklist for Facilitating Return to work through Early Specialist Health-based interventions FRESH)**

| **FRESH vocational rehabilitation (VR) Intervention Components**  ***Words in italics are related to frequency of intervention*** | **Extent of delivery (always, often, sometimes, seldom, never)**  **Also consider content, coverage, frequency, duration** | **Moderators - +ve & -ve**  **Also consider participant responsiveness, resources, recruitment, context, comprehensiveness of policy description, strategies to facilitate implementation, quality of delivery** |
| --- | --- | --- |
| 1. Occupational therapist (OT) and case manager (CM) interventions are tailored to each participant and explicitly work-focused (content form will capture detail). However, they include the following essential intervention components (numbers 13-19). |  |  |
| 1. Assessing the impact of traumatic brain injury (TBI) on function and work role and providing individualised education to participant with a TBI (PwTBI), family and employer. *Starts <10 days post recruitment.* |  |  |
| 1. Developing strategies for the PwTBI to explain the effects of their TBI to others. |  |  |
| 1. Developing strategies to manage TBI effects in everyday life and work/study. |  |  |
| 1. Assessing the work/study role, work duties/functions, work/job demands. |  |  |
| 1. Supporting the PwTBI in seeking and accepting feedback about their (work) function. |  |  |

| 1. Exploring options for returning to work (RTW). |  |  |
| --- | --- | --- |
| 1. Retraining or practicing work skills/functions. |  |  |
| 1. OT negotiates a graded RTW. The OT then monitors the PwTBI *at least weekly in the first 1-2 weeks; weekly for the next 4 weeks; then every 2 weeks for the following 8 weeks; then every 6-8 weeks unless decided otherwise by PwTBI.* |  |  |
| 1. CM supports PwTBI and family with work-related issues and provides feedback, e.g. by meeting before and after workplace review sessions. |  |  |
| 1. OT and CM support PwTBI to develop and maintain coping skills to help them deal with risks to job retention. *OT monitors these every 4-8 weeks once the graded RTW plan is complete.* |  |  |
| 1. OT formulates vocational rehab plan on the basis of the assessments results. |  |  |
| 1. All planning is done in consultation with the PwTBI. |  |  |
| 1. OT/CM informs other professionals and care providers about the VR plans. |  |  |
| 1. CM ensures that non-work focused activities are coordinated and continue. |  |  |
| 1. OT contacts PwTBI *every 1-2 weeks except in cases where more frequent contact is needed.* |  |  |
| 1. CM reviews participant *every 6-8 weeks except in cases where more frequent contact is needed*. |  |  |
| 1. At the end of the workplace intervention OT/CM writes thank you letter to employer and cc participant’s GP. |  |  |

| Adherence  Content – was each of the intervention components implemented as planned?  Coverage – what proportion of the target group participated in the intervention?  Frequency – how often was the intervention provided?  Duration – how long was either each intervention session and/or the overall duration of the intervention? | Moderating factors  Participant responsiveness – How were the participants engaged with the intervention service? How satisfied were the participants and how did the participants perceive the outcomes and relevance of the intervention?  Recruitment – what procedures were used to attract participants? What constituted barriers to maintaining involvement of individuals?  Context – what factors at the political, economical (resources, money and time) or organisational level affected the implementation?  Comprehensiveness of policy description – How specific is the intervention description?  Strategies to facilitate implementation – what strategies were used to support implementation? How were these strategies perceived by staff?  Quality of delivery – how was the quality of the intervention components? |
| --- | --- |
