# Supplementary material for: Developing a method to assess fidelity to a complex vocational rehabilitation intervention in the FRESH trial: a feasibility study
Source: Pilot Feasibility Stud. 2022 Jul 29;8:160. doi: 10.1186/s40814-022-01111-2 (PMC9335967; doi:10.1186/s40814-022-01111-2)
Supplement: Supplementary file 6 — Additional file 6. [file 40814_2022_1111_MOESM6_ESM.docx]

## Additional File 6: Mentoring CRF

| **FRESH Peer/ Mentor Support Form** | | | | | |
| --- | --- | --- | --- | --- | --- |
| **Therapist Name:** |  | | **Date:** |  | |
| **Mentor Name:** |  | | **Time spent:** |  | |
| **Method used:** |  | | | | |
| **Organisation:** |  | | | | |
| **Topic/issue notes** | | **Agreed Action (if applicable) notes (action by whom and date required)** | | | |
| Recruitment | |  | | | |
| Research Documentation | |  | | | |
| Research process/Implementation | |  | | | |
| ESTVR intervention/ Client-related | |  | | | |
| Other including serious adverse events | |  | | | |
| **Agreed as correct content.** |  | **Next session date and method:** | | |  |
